# ESC reverses epithelial mesenchymal transition induced by transforming growth factor-β via inhibition of Smad signal pathway in HepG2 liver cancer cells

**DOI:** 10.1186/s12935-015-0265-2

**Published:** 2015-12-10

**Authors:** Xiao-Ni Liu, Shuang Wang, Qing Yang, Ya-Jie Wang, De-Xi Chen, Xiao-Xin Zhu

**Affiliations:** Beijing Institute of Hepatology and Beijing YouAn Hospital, Capital Medical University, No 8 Xi Tou Tiao, You An Men Wai, Feng Tai Qu, Beijing, 100069 China; Institute of Chinese Materia Medica, China Academy of Chinese Medical Sciences, No 16 Nan Xiao Jie, Dong Zhi Men Nei, Dong Cheng Qu, Beijing, 100700 China

**Keywords:** Hepatocellular carcinoma, *Stellera chamaejasme* L., Transforming growth factor, Metastasis, Epithelial mesenchymal transition, Smad signaling pathway

## Abstract

**Background:**

Epithelial mesenchymal transition (EMT) mediated by TGF-β pays an important role in malignant tumor acquired abilities of migration and invasion. Our previous study showed that the extract of *Stellera chamaejasme* L. (ESC) was against proliferation of a variety of tumor cells, but there were no studies in the effects of ESC on EMT in tumor cells. In this study, TGF-β was adopted to induce EMT in HepG2 cells and the influence of ESC on EMT was observed.

**Methods:**

MTT assay was used to observe the cell viability. Wound healing assay and transwell assay were used to observe the migration and invasion activities. Western blot and immunofluorescence methods were used to observe the expression of proteins.

**Results:**

We found that HepG2 cells induced by TGF-β showed mesenchymal morphology, down-regulation of epithelial marker E-cadherin and up-regulation of mesenchymal marker Vimentin, indicating that TGF-β could mediate epithelial mesenchymal induction in HepG2 cells. ESC could reverse the mesenchymal morphology and regulate expressions of marker proteins in HepG2 induced by TGF-β and significantly inhibit TGF-β induced HepG2 cell migration and invasion. We further found that ESC could also significantly depress Smad2 phosphorylation and nuclear translocation, and ESC had coordination with SB432542, a specific inhibitor of TβRI kinases.

**Conclusions:**

These results suggested that the ESC could reverse epithelial mesenchymal transition induced by TGF-β via inhibition Smad2 signaling pathway.

## Background

In recent years, it is found that epithelial mesenchymal transition (EMT) is an important biological process for malignant tumor cells to obtain migratory and invasive ability and a key initiative step of invasion and metastasis in tumors. EMT is characterized by up-regulation of mesenchymal markers (such as Vimentin) down-regulation of epithelial markers (such as E-cadherin) [1], and loss of cell–cell adhesion, which enables tumor cells to dissociate and migrate from the primary tumor [2]. Because EMT is closely related to the proliferation, metastasis and prognosis of malignant tumor, it has become an important hot spot for pharmacological studies on tumors [3]. Transforming growth factor (TGF-β) is one of the most important signal molecular that can initiate the EMT process [4]. During TGF-β-mediated EMT, TGF-β initiates responses by contacting two types of transmembrane serine/threonine kinases called receptors type I and type II, promoting activation of the type I by the type II kinase. The activated type I receptor then propagates the signal to the nucleus by phosphorylating Smad2 and Smad3. Once phosphorylated, Smad2 and Smad3 associate with the shared partner Smad4 and the complexes accumulate in the nucleus where they regulate the expression of TGF-β target genes through cooperative interactions with transcriptional partners, which is process of the classical Smad-dependent signaling pathway that TGF-β induced [5, 6]. Many studies showed that a variety of *Stellera chamaejasme* L. extracts or monomers had anti-tumor activities and could induce apoptosis of tumor cells [7–10]. Our previous studies also got the same results [11, 12], but there were no experiments on its anti-metastasis effects. In this study, we further observed the effects of ESC on TGF-β-mediated EMT and classical Smad-dependent signaling pathway in HepG2 liver cancer cells.

## Methods

### Cell line and cell culture

HepG2 liver cancer cell lines were preserved in Beijing Institute of Hepatology. HepG2 cells were cultured in DMEM medium (Gibco, Grand Island, NY, USA) supplemented with 10 % fetal bovine serum (China Hangzhou Sijiqing Biological Technology Co., Ltd) and maintained at 37 °C in a humidified incubator with 5 % CO_2_.

### Reagents and antibodies

Process of ESC (Extract of *Stellera Chamaejasme* L.) and determination of part components of ESC was provided in another paper [11]. Trypsin-ethylene-diaminetetraacetic acid (EDTA) and DMEM medium were purchased from Gibco (Grand Island, NY, USA);3-(4,5-dimethyl-2-thiazolyl)-2,5-diphenyl-2-H-tetrazolium bromide (MTT), dimethyl sulfoxide (DMSO) and SB431542 were provided by Sigma Chemical Co. (St. Louis, MO, USA); TGF-β was from R&D Systems (Miniieapolis, MN, USA); E-cadherin, Vimentin and β-actin primary monoclonal antibody were purchased by Abcam Ltd (Cambridgem MA, USA); Matrigel was from BD Biosciences (Los Angeles, CA, USA); Crystal violet was from Beijing Solarbio Science and Technology Co., Ltd; Smad and p-Smad primary monoclonal antibody were from Cell Signal Technology, Inc (Beverly, MA, USA).

### MTT assay

Cells in the logarithmic growth phase were plated in 96-well plates in a seeding density of 5000 cells/well and incubated in a 37 °C incubator with 5 % CO_2_ overnight. After cells were treated with ESC (final concentration was respectively, 100, 50, 25, 12.5, 6.25, 3.125, 1.562, 0 μg/mL) for 24, 48, 72 h, the culture medium in each well was abandoned, incubating with 0.5 g/L MTT 100 μL for 4 h. Then each well was added with 150 μL DMSO and vibrated for 10 min, and absorbance of each well was detected with microplate reader (ELX800 type, BIO-TEX Instruments, INC, Winooski, VT, USA) at the 490 nm wavelength. The inhibition rate (IR) was calculated as follows: IR (%) = (1 − OD_treatment_/OD_control_) × 100 %. Then half-maximal inhibitory concentration (IC_50_) was determined by logistic method.

### Scattering assay

Cells (2 × 10^4^) were seeded in each well of 24-well plate and incubated at 37 °C with 5 % CO_2_ overnight. Cells were pretreated with indicated concentration of ESC for the appropriated time and then TGF-β was added to each well with the final concentration of 5 ng/mL. Cells were incubated at 37 °C with 5 % CO_2_ for 24 h. Representive photographs were taken using an inverted microscope (Olympus, Japan).

### Wound healing assay

Cells (5 × 10^5^) were plated in a 6-well plate (three lines were drawn at the external bottom of each well) and incubated in a 37 °C incubator with 5 % CO_2_ overnight to form a confluent monolayer. The monolayers were scratched vertically to the lines by a plastic tip and washed by PBS to remove cell debris. Indicated concentrations of ESC and 5 ng/mL TGF-β were then added to each well, and the plates were incubated at 37 °C incubator with 5 % CO_2_ for 24 h. Photographs of wound closure were taken at the intersection point of lines and scratches at the point time of 0 and 24 h by the inverted microscope and the distance of wound closure was measured by Photoshop 8.0 software.${\text{Relative}}\;{\text{closure rate }}(\% \text{)} = 1 - \frac{{{\text{Distance of treatment group at }}0{\text{ h}} - {\text{Distance of treatment group at }}24{\text{ h}}}}{{{\text{Distance of control group at }}0{\text{ h}} - {\text{Distance of control group at }}24{\text{ h}}}}$

### In vitro invasion assay

The matrigel invasion experiment was analyzed in 24-well transwell plates. 25 μL matrigel matrix was resolved at 4 °C overnight and coated on the transwell insert membrane (Corning Incorporated). After the inserts were incubated at 37 °C for 30 min, 2 × 10^4^ cells in 100 μL of DMEM medium with 1 % BSA and different concentrations of ESC were added to the top chamber and 500 μL of 10 % serum-containing DMEM and 5 ng/mL TGF-β were added in the bottom chamber. The cells were then incubated at 37 °C with 5 % CO_2_ for 24 h. After incubation, the medium was removed, and non-invading cells were scrubbed by a wet cotton swab. The invading cells were washed by PBS for three times and fixed by 4 % paraformaldehyde for 15 min. Fixed cells were washed three times by PBS and stained by 0.1 % crystal violet in PBS for 10 min. Excess stain was washed by distilled water for three times. The invading cells were counted in five random fields using an inverted microscope.

### In vitro migration assay

The methods of migration assay were same to the invasion assay, but the transwell insert membranes were not coated by matrigel.

### Western blot analysis

Cells were seeded in 100 mm tissue culture dishes at the density of 2 × 10^6^ cells per dish and incubated for overnight. Cells were then treated with various agents as indicated in figure legends, then washed with ice-cold PBS and harvested in 400 μL of cell lysis buffer. The protein concentrations of lysates were determined using the bicinchonininc acid method. Cell lysates (40 μg per lane) were separated using 10 % SDS-PAGE and transferred electrophoretically to polyvinylidenedifluoride membrane. Membranes were blocked with tris-buffered saline/0.1 % tween 20 containing 5 % bovine serum albumin and then incubated overnight at 4 °C with primary antibodies (1:1000). Membranes were washed three times with TBST and incubated for 1 h at room temperature with the appropriate secondary antibody conjugated to goat anti-rabbit horseradish peroxidase (1:2000). Membranes were then washed and immunoreactive band were developed with ECL and visualized by autoradiography. Protein loading was normalized using β-actin antibody. Gray-scale analysis of protein bands was performed using image software.

### Immunofluorescence analysis

Cells were seeded into 24-well plates and treated as described above. Cells were fixed with 4 % formaldehyde for 30 min, washed with PBS, blocked with 5 % BSA for 30 min at room temperature, and then stained with anti-human primary antibody (1:100) at 4 °C overnight. Cells were incubated with anti-rabbit-FITC secondary antibody (1:500) for 2 h at 4 °C, and then washed with PBS. Cells were then incubated for 10 min at room temperature with DAPI to stain nuclei, washed twice with PBS, and observed using an inverted fluorescence microscope (Olympus, Japan).

### Statistical analysis

All the data were expressed as the mean ± SD. The results were subjected to the one way ANOVA test using SPSS software (17.0 version).

## Results

### TGF-β induced EMT in HepG2 cells

**Fig. 1:**
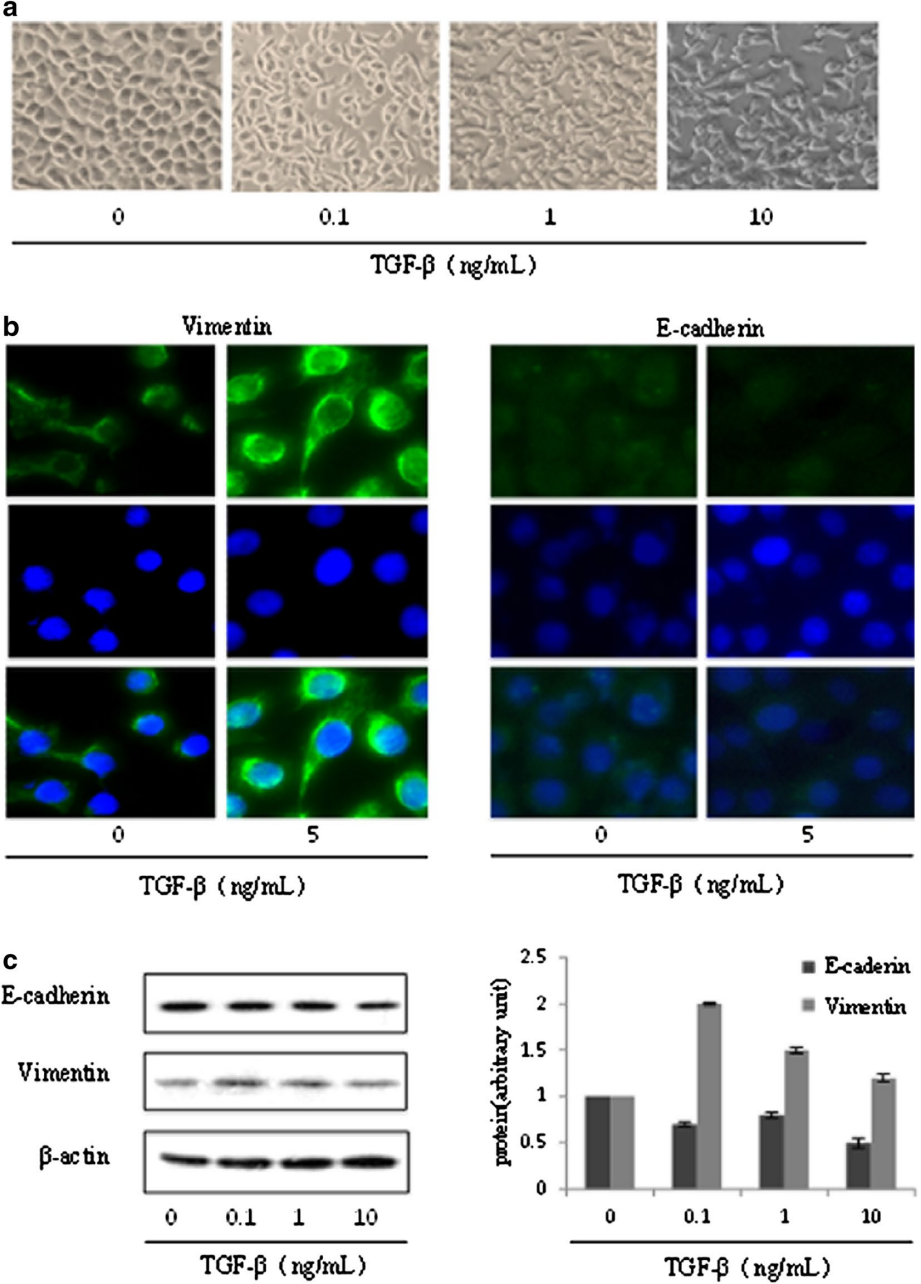
TGF-β induced EMT in HepG2 cells. **a** Changes of cell morphology were assessed following treatment of HepG2 cells with various concentrations (0.1–10 ng/mL) of TGF-β for 24 h. The magnification is 100 times. **b** The expressions of E-cadherin and Vimentin were determined with immunofluorescence method in HepG2 cells treated with TGF-β (5 ng/mL) for 24 h. The magnification is ×400. **c** The expressions of E-cadherin, and Vimentin were measured with western blot analysis in HepG2 cells treated with various concentrations (0.1–10 ng/mL) TGF-β for 24 h

First, the optimal concentrations of TGF-β to initiate the EMT in HepG2 cells were determined. Changes of cell morphology were observed after treatment with various concentrations (0.1–10 ng/mL) of TGF-β for 24 h. HepG2 cells were subjected to morphological changes under exposure to various concentrations of TGF-β. HepG2 cells showed a classical cobblestone epithelial morphology in the absence of TGF-β, but after stimulation with various concentrations of TGF-β for 24 h, the cells showed a fibroblast-like morphology and cell–cell adhesion also reduced (Fig. 1a). The expressions of epithelial phenotype marker, E-cadherin, and mesenchymal phenotype marker, Vimentin, were also determined after treatment with various concentration of TGF-β for 24 h in HepG2 cells. TGF-β decreased the E-cadherin and induced the Vimentin expression in HepG2 cells (Fig. 1b, c). These results suggested that TGF-β could induce EMT in HepG2 cells.

### The cytotoxic effect of ESC on HepG2 cells

**Fig. 2:**
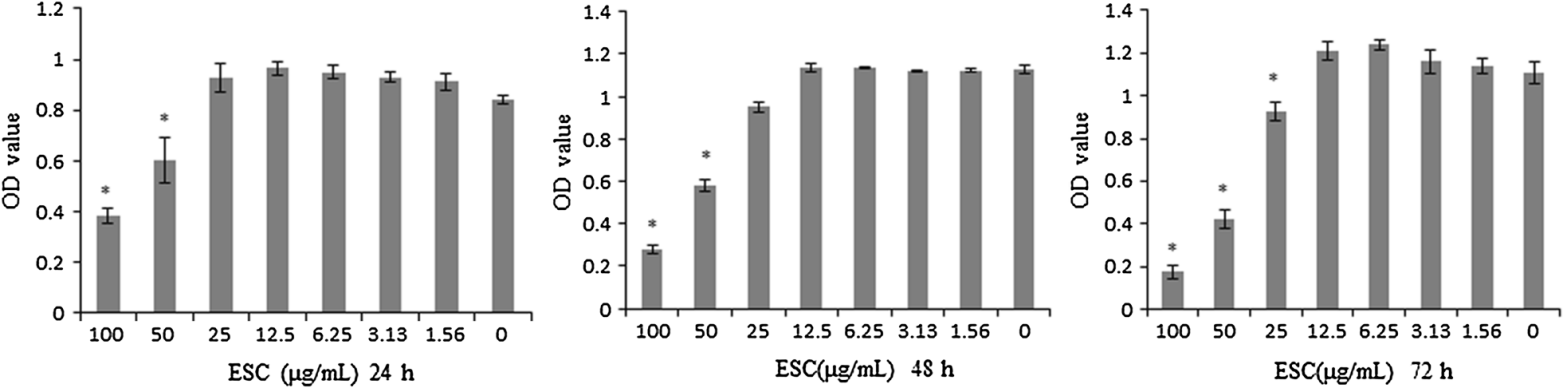
The inhibitory effect of ESC on HepG2 cells. The OD values of HepG2 cells treated with various concentrations ESC (0–100 μg/mL) were determined using MTT assay (*p < 0.05 vs control group)

The dosage of ESC for EMT experiments must be determined to avoid the influence of anti-proliferation of ESC. Cytotoxicity of ESC was evaluated in HepG2 cells using MTT assay. Doses of ESC up to 12.5 μg/mL (0, 1.56, 3.13, 6.25) exhibited no significant inhibition on HepG2 cells, whereas higher doses of ESC (>12.5 μg/mL) significantly inhibited HepG2 cell viability in HepG2 cells (Fig. 2). IC50 of ESC on HepG2 cells for 24, 48, 72 h were respectively, 85.99, 59.75 and 54.57 μg/mL. Therefore, the doses less than 12.5 μg/mL were chosen for following EMT experiments.

### ESC reversed cell scattering induced by TGF-β in HepG2 cells

**Fig. 3:**
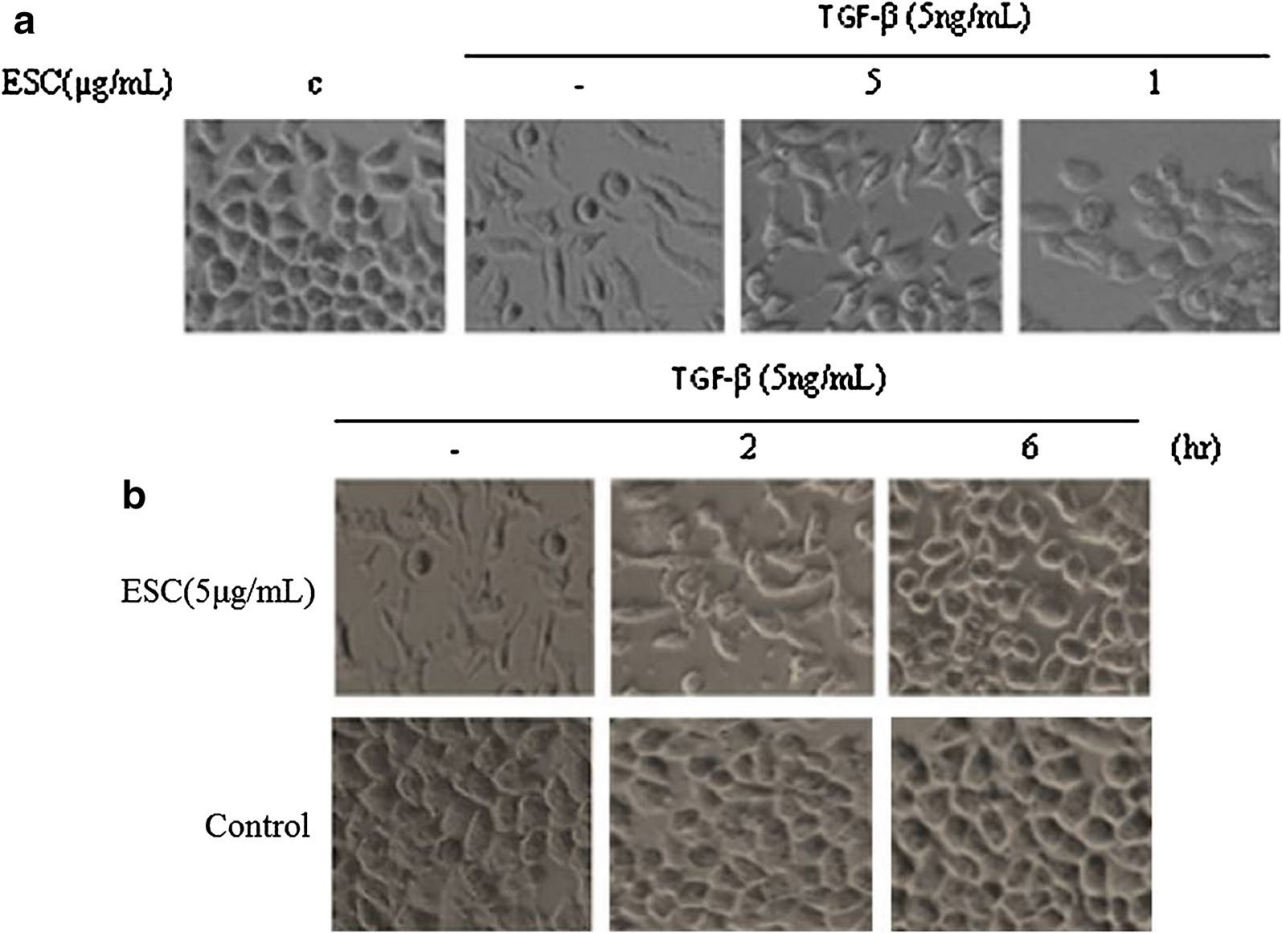
ESC reversed cell scattering induced by TGF-β in HepG2 cells.** A** Different concentration ESC (1–5 μg/mL) on cell scattering induced by TGF-β for 24 h. The magnification is ×200.** B** HepG2 cells treated with 5 μg/mL ESC for varying periods (2–6 h) prior to TGF-β addition. Vehicle (medium containing 0.2 % DMSO) was used to be as parallel control. The magnification is ×200. Note: **c** in this figure and other figures means vehicle control

The TGF-β induced EMT was characterized by cell scattering (Fig. 1). To make out whether ESC could influence TGF-β-induced cell scattering, HepG2 cells were stimulated with TGF-β (5 ng/mL) before treated with ESC. In order to eliminate the interference of proliferation inhibition of ESC on its block-scattering, we chose low toxicity concentrations (<12.5 μg/mL) of ESC for the further experiments. HepG2 cells were pretreated with various concentrations of ESC (1–5 μg/mL) for 2 h before stimulated with TGF-β for 24 h. The results showed that 5 and 1 μg/mL ESC blocked this scattering significantly (Fig. 3A). We pretreated HepG2 cells with 5 μg/mL ESC for varying periods from 2 to 6 h before TGF-β addition and time-dependent reversing effect of ESC was showed (Fig. 3B). These results showed that ESC could inhibit the cell scattering induced by TGF-β in HepG2 cells.

### ESC reversed EMT induced by TGF-β in HepG2 cells by down-regulation of Vimentin and up-regulation of E-cadherin

**Fig. 4:**
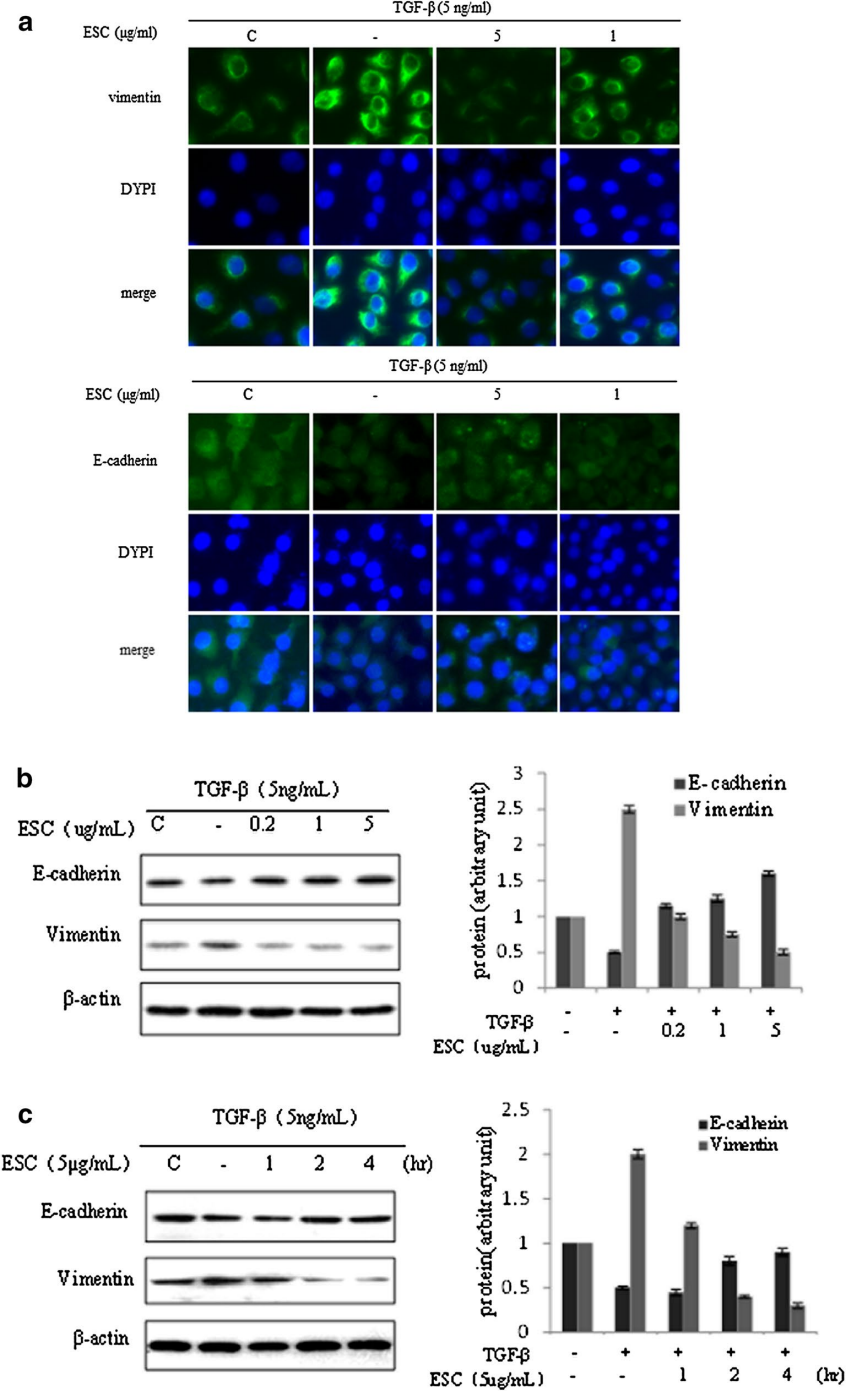
ESC reversed EMT induced by TGF-β in HepG2 cells by down-regulation of Vimentin and up-regulation of E-cadherin. **a** Expressions of Vimentin and E-cadherin in HepG2 cells pretreated with different concentrations (1–5 μg/mL) of ESC prior 2 h to TGF-β (5 ng/mL) incubation for 24 h with immune-fluorescence assay. The magnification is ×400. **b** Western blot results of Vimentin and E-cadherin in HepG2 cells pretreated with different concentrations (0.2–5 μg/mL) of ESC prior 2 h to TGF-β (5 ng/mL) incubation for 24 h. **c** Western blot results of Vimentin and E-cadherin in HepG2 cells pretreated with 5 μg/mL ESC for different hours (1–4 h) prior to 5 ng/mL TGF-β stimulation. Western blot data presented were representative of those obtained in at least 3 separate experiments. The value of the control cells was set to 1

Pretreated with ESC (0.2–5 μg/mL) for 2 h in HepG2 cells significantly inhibited up-regulation of Vimentin and down-regulation of E-cadherin induced by 5 ng/mL TGF-β for 24 h (Fig. 4a, b). HepG2 cells were pretreated with 5 μg/mL ESC for different hours (1–4 h) prior to 5 ng/mL TGF-β stimulation, the time-dependent effects of ESC were showed (Fig. 4c). These results suggested that ESC reversed marker proteins changes of EMT induced by TGF-β in HepG2 cells.

### ESC inhibited the TGF-β-induced cell migration and invasion

**Fig. 5:**
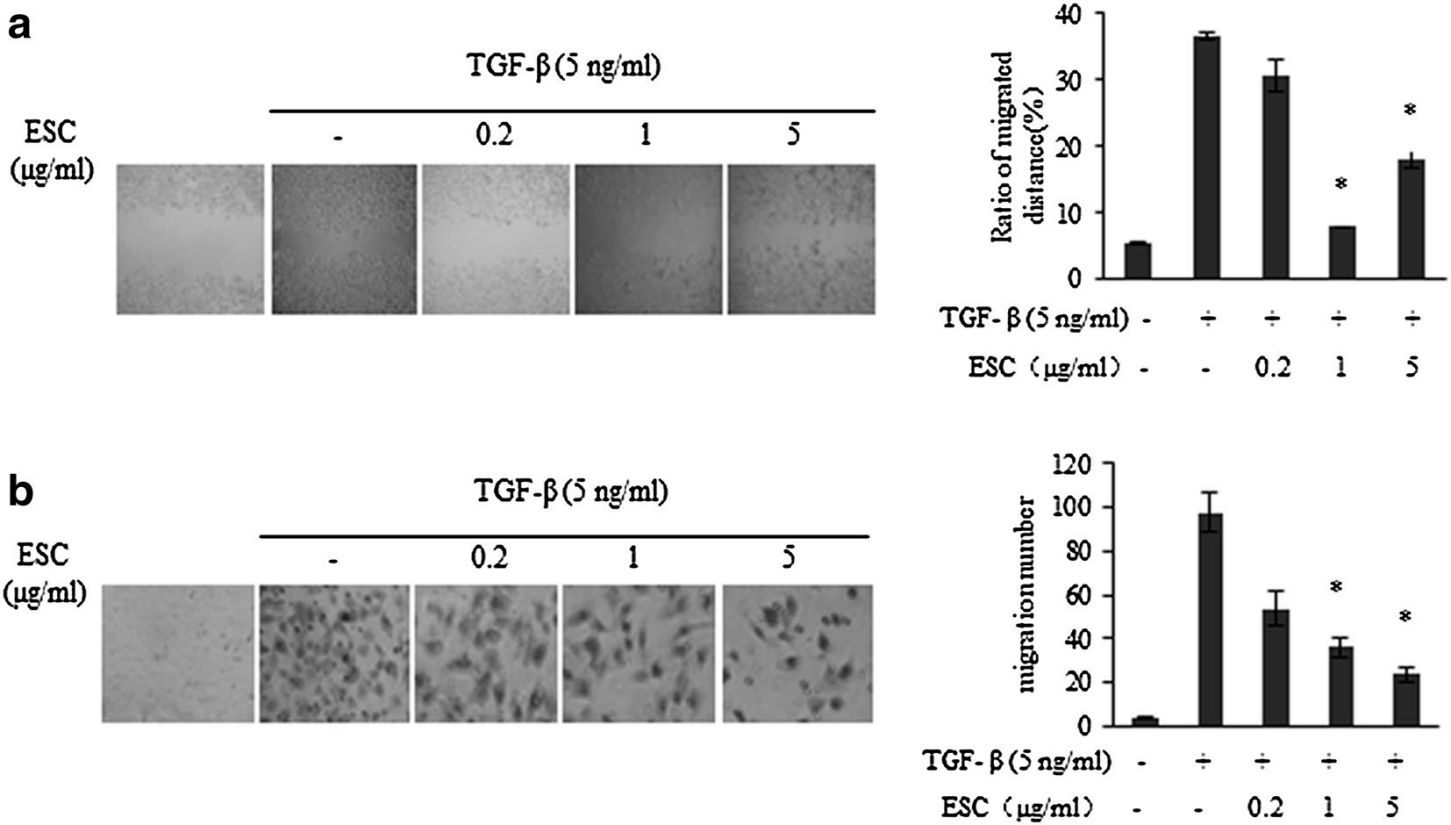
Effects of ESC on TGF-β-induced cell migration and invasion. **a** Effect of ESC on TGF-β induced cell migration with wound healing assay. ESC (0.2–5 μg/mL) significantly inhibited TGF-β induced cell motility (*p < 0.05 vs control group).The magnification is 40 times. **b** Effect of ESC (0.2–5 μg/mL) on TGF-β induced cell invasion with transwell assay. ESC (0.2–5 μg/mL) significantly inhibited TGF-β-induced cell invasion (*p < 0.05 vs control group). The magnification is ×100

We also further examined whether ESC affected TGF-β-induced cell migration and invasion. Our results showed that TGF-β significantly induced cell migration and ESC (0.2–5 μg/mL) could notably block this migration (Fig. 5a). The same results were observed with transwell assay and the results were not showed. A modified invasion assay with transwell method was also performed to further determine whether ESC blocked TGF-β-induced invasion. ESC (0.2–5 μg/mL) obviously decreased the number of TGF-β induced invasive cells (Fig. 5b). These results suggested that ESC inhibited the TGF-β-induced cell migration and invasion in HepG2.

### ESC inhibited TGF-β-initiated Smad signaling pathway

**Fig. 6:**
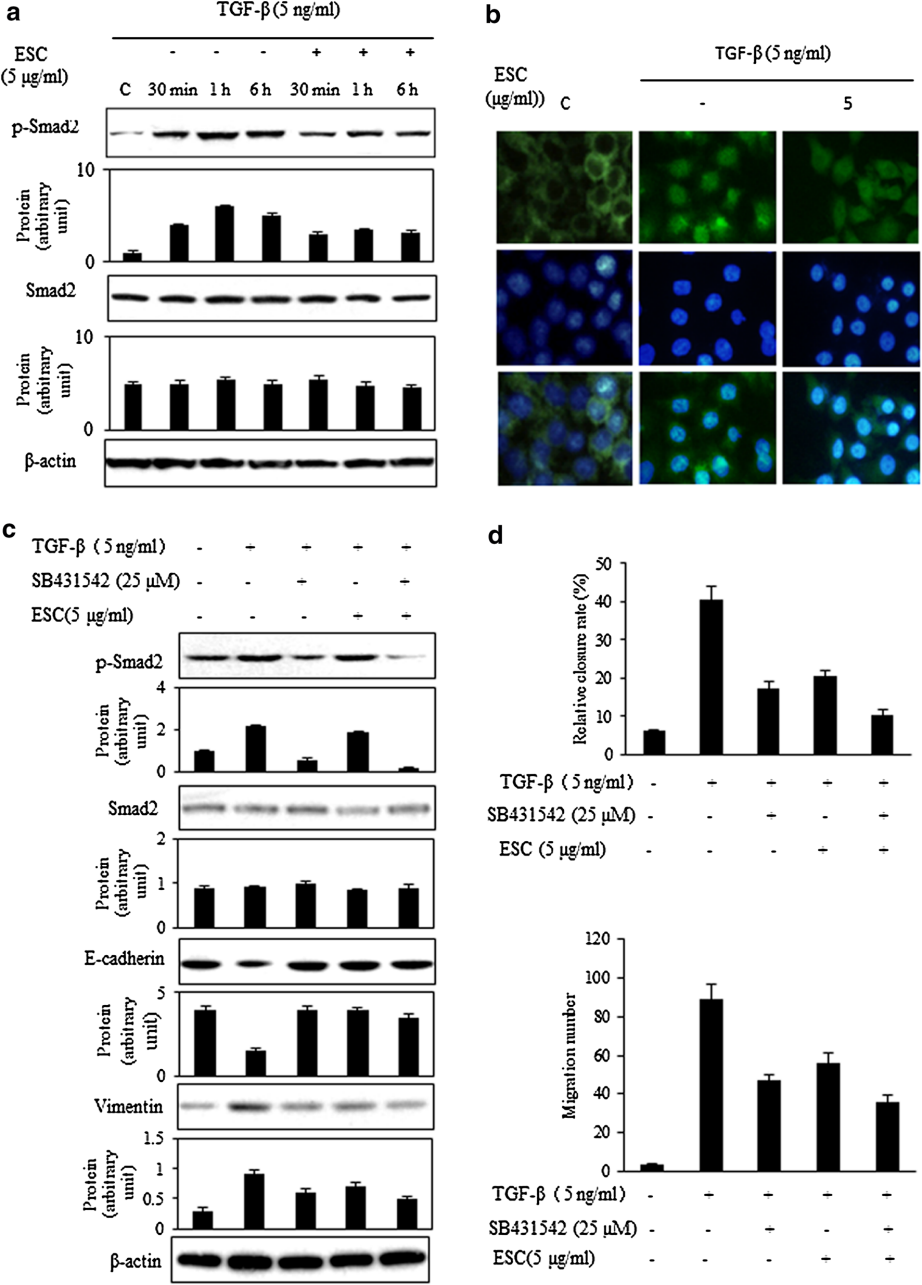
ESC inhibited TGF-β-induced Smad signaling pathway. **a** Pretreatment with 5 μg/mL ESC for 2 h significantly suppressed the expression of phosphorylation of Smad2 induced by TGF-β for 0.5–6 h, but no influence on expression of Smad2. **b** Pretreatment of 5 μg/mL ESC for 2 h could inhibit the nucleus translocation induced by TGF-β in hepG2 cells. The magnification is 200 times. **c** 5 μg/mL ESC and 25 μM SB432542 significantly reversed EMT proteins induced by TGF-β, inhibiting expression of phosphorylation of Smad2 and Vimentin, enhancing expression of E-cadherin and the coordination of ESC and SB432542 was showed. **d** 5 μg/mL ESC and 25 μM SB432542 could inhibited cell migration and invasion induced by TGF-β and also showed coordination effects

Since TGF-β could induce the Smad2 phosphorylation, we tried to figure out whether ESC could inhibit this initiation. We found that 5 ng/mL of TGF-β induced Smad2 phosphorylation within 0.5–6 h, and the level of Smad2 phosphorylation reached a maximum between 30 and 60 min after treatment but total Smad2 expression was not be affected during the whole stimulation period. Pretreatment with 5 μg/mL ESC for 0.5–6 h significantly inhibited the expression of phosphorylation of Smad2 induced by TGF-β, but no influence on the whole smad2 expression (Fig. 6a). Our results found that 5 ng/mL TGF-β could induce Smad2 nucleus translocation and pretreatment of ESC (5 μg/mL) for 2 h could reverse this nucleus translocation in hepG2 cells (Fig. 6b). These results suggested that ESC could inhibit TGF-β-induced Smad2 phosphorylation and Smad2 Nuclear import. The western blot analysis revealed that TGF-β induced smad2 phosphorylation, down-regulated the E-cadherin and up-regulated the Vimentin. SB431542 (25 μM) could reverse these effects of TGF-β in HepG2 cells. The ESC (5 μg/mL) also could inhibit Smad2 phosphorylation, up-regulate E-cadherin and down-regulate Vimentin induced by TGF-β. The reversing effects of copretreatment with ESC (5 μg/mL) and SB432542 (25 μM) on phosphorylation of smad2 and Vimentin were more potent than pretreatment with SB431542 or ESC alone (Fig. 6c). Cell migration and invasion were significantly inhibited by treatment with SB431542 and ESC, copretreatment with ESC and SB432542 showed more potent inhibition on cell migration and invasion induced by TGF-β than treatment with SB431542 or ESC alone (Fig. 6d). These results suggested that ESC could reversing EMT induced by TGF-β and this reversing effect might be related to its inhibition of Smad signaling pathway.

## Discussion

Hepatocellular carcinoma is an extreme malignant tumor, and early metastasis is one of the important reasons for its poor prognosis. EMT is an important step in the invasion and metastasis of many cancers, and TGF-β induces progression of cancer through EMT [13, 14]. Many researches confirmed that high expression or activation of TGF-β in metastatic tumors [15, 16]. So, as a classic model of EMT induced by TGF-β, it was widely used and we made this model in HepG2 cells to mimic the metastasis in vitro. Our experiment results showed that HepG2 cells were sensitive to the different concentration of TGF-β (0.1–10 ng/mL) and TGF-β not only changed the morphology but also the molecular markers of the HepG2 cells. HepG2 cells induced by TGF-β appeared the fibroblast-like morphology, with down-regulation of the E-cadherin and up-regulation of Vimentin, which was consistent with other reports [17]. These results suggested that TGF-β could induce EMT in HepG2 cells.

ESC, the extract of *Stellera Chamaejasme* L., inhibited proliferation and induced apoptosis in various tumor cells by activation apoptotic death receptor signaling pathway in our previous researches [11, 12]. In this study, we found that EMT induced by TGF-β in HepG2 cells could be reversed by ESC (doses less than 12.5 μg/mL were used to avoid the influence of anti-proliferation of ESC). The results showed ESC (1–10 μg/mL) could reverse the cell scattering induced by TGF-β and this effect appeared time-dependent style. To further clarify whether ESC inhibition of TGF-β-induced scattering in HepG2 cells resulted from dysregulation of EMT related proteins, influence of ESC on E-cadherin and Vimentin expression was observed. E-cadherin (epithelial marker protein) is a well-studied member of the cadherin family. In epithelial cells, E-cadherin-containing cell-to-cell junctions are often adjacent to actin-containing filaments of the cytoskeleton [18]. Loss of E-cadherin function or expression has been implicated in cancer progression and metastasis. E-cadherin downregulation decreases the strength of cellular adhesion within a tissue, resulting in an increase in cellular motility [19]. Vimentin is a type III intermediate filament (IF) protein that is expressed in mesenchymal cells. Vimentin is the major cytoskeletal component of mesenchymal cells. Because of this, Vimentin is often used as a marker of mesenchymally-derived cells or cells undergoing an epithelial mesenchymal transition during both normal development and metastatic progression [20, 21]. We examined the expressions of these two marker proteins in HepG2 cells with immunofluorescence and western blot assay. ESC (0.2–5 μg/mL) could reverse the changes of two marker proteins in hepG2 cells induced by TGF-β which dedicated that ESC could decrease cellular motility and metastasis. The results of wound closure and transwell assay further confirmed this inhibition effect of ESC in migration and invasion induced by TGF-β. These data suggested that ESC could restrain the EMT mediated by TGF-β.

How does the ESC reverse TGF-β-induced EMT in hepG2 cells? Smad-dependent signaling was classical pathway mediated by TGF-β. TGF-β superfamily ligands bind to a type II receptor, which recruits and phosphorylates a type I receptor. The type I receptor then phosphorylates receptor-regulated Smad2/3 which can bind the Smad4. P-Smads/Smad4 complexes accumulate in the nucleus where they act as transcription factors and participate in the regulation of EMT target gene expression [22, 23]. Under resting state, Smad2 is unphosphorylated and retain in the cytoplasma. Upon activation of TGF-β, Smad2 is phosphorylated and undergoes dimerization with Smad3, thus permitting its translocation into nucleus [24]. Our results showed that ESC could inhibit the up-regulation of p-Smad2 but did not influence the expression of Smad2. The subcellular location results showed that ESC could inhibit the Smad2 nuclear translocation. These results suggested that ESC really could reverse TGF-β induced EMT by regulating Smad signal pathway.

In Smad signal pathway, which was the key regulating point of ESC in reversing EMT induced by TGF-β in hepG2 cells? We use a specific inhibitor of TβRI kinases, SB432542 [25] to inhibit TGF-β-induced phosphorylation of Smad2 [25]. We found that blocking the function of TβRI kinases with SB432542 resulted in inhibition of Smad2 phosphorylation, up-regulation of E-cadherin, down-regulation of Vimentin, with the decreasing activities of migration and invasion in hepG2 cells induced by TGF-β. ESC not only had same effects to SB432542, but also enhanced these effects of SB432542. These results suggested that ESC might be a potential inhibitor of TβRI kinases which might be the key regulating point of ESC in reversing EMT induced by TGF-β in hepG2 cells.

In summary, our observations showed that ESC could reverse the EMT mediated by TGF-β in hepG2 liver cells via inhibition of Smad signaling pathway. ESC may have good prospects in the prevention and treatment of hepatocellular carcinoma metastasis.
